# Pilot study of an arterial enhancement fraction-based model for progression prediction in HCC undergoing chemoembolization

**DOI:** 10.3389/fonc.2025.1489450

**Published:** 2025-02-19

**Authors:** Bin Chai, Dongqiao Xiang, Guofeng Zhou, Chuansheng Zheng

**Affiliations:** ^1^ Department of Radiology, Union Hospital, Tongji Medical College, Huazhong University of Science and Technology, Wuhan, China; ^2^ Hubei Province Key Laboratory of Molecular Imaging, Wuhan, China

**Keywords:** hepatocellular carcinoma, transarterial chemoembolization, prognosis, multidetector computed tomography, quantitative evaluation

## Abstract

**Objective:**

To develop a prognostic model including arterial enhancement fraction of residual tumor (AEF-RT) for predicting progression-free survival (PFS) in hepatocellular carcinoma (HCC) patients after drug-eluting beads transarterial chemoembolization (DEB-TACE).

**Materials and methods:**

Between March 2019 and March 2024, 111 HCC patients undergoing DEB-TACE were randomly allocated to a training cohort and a validation cohort in a 7:3 ratio. LASSO regression was applied in the training cohort to identify risk factors for recurrence, which were subsequently used to construct the Cox model. Model performance was assessed using the concordance index (C-index, where 0.5 indicates non-informative discrimination and 1 represents perfect discrimination) and Brier score (ranging from 0 to 1, 0 indicating higher calibration) and was compared with five existing prognostic models.

**Results:**

The final model, termed ADMAN model, incorporated AEF-RT, Diameter, Margin appearance, Aspartate transaminase, and Neutrophil-to-lymphocyte ratio. High-risk patients defined by ADMAN had 4.69 times greater progression risk than low-risk ones in the training cohort (*p* < 0.001) and 3.52 times greater in the validation cohort (*p* = 0.005). The C-index of ADMAN (0.75) was significantly higher than that of other models in the training cohort (*p* < 0.05 for all) and remained significantly higher than three of them in the validation cohort [0.71 vs. 0.55 (*p* = 0.041), 0.54 (*p* = 0.033), 0.53 (*p* = 0.004)]. The ADMAN model showed a significantly lower Brier score than that of other models at 6 months and 12 months in the training cohort (*p* < 0.05 for all). In the validation cohort, the ADMAN model remained to have significantly lower Brier score than the four models (*p* < 0.05) at 6 months, while it had significantly lower score than one model at 12 months.

**Conclusions:**

The AEF-based model may be a promising tool for progression risk stratification in HCC patients undergoing DEB-TACE. Further external validation in independent cohorts with larger sample sizes is necessary to confirm the robustness of the ADMAN model.

## 1 Introduction

Transarterial chemoembolization (TACE) is a well-established treatment strategy for patients with hepatocellular carcinoma (HCC) who are not eligible for curative treatments (1). Nevertheless, the objective response rates 6 months after TACE range from 27% to 76% and 70% to 80% in patients who eventually die due to tumor progression (2). Transitioning from TACE to systemic therapies is advisable for patients unlikely to benefit from repeated embolization before liver function deteriorates. Therefore, it is imperative to develop methods for estimating individualized treatment efficacy. Several studies have suggested that 18F-fluorodeoxyglucose uptake, clinical characteristics, tumor radiological features, and certain serum biomarkers may serve as promising indicators for tumor progression after TACE (3–7). However, few studies have developed multivariate algorithms combining clinical and imaging findings to predict progression for individual patients.

A key element that influences the aggressiveness of HCC is tumor neo-angiogenesis, the process of developing new capillary blood vessels that results in tumor vascularization. The presence of high vascularity typically indicates aggressive tumor behavior and is linked to poorer clinical outcomes. CT perfusion may reveal tumor aggressiveness and predict prognosis based on tumor vascularity (8). However, the application of such a technique is limited by the high radiation exposure. Arterial enhancement fraction (AEF) is defined as the ratio of the absolute increment of attenuation in the arterial phase to that of the portal venous phase: AEF = [(HU_A_ − HU_U_)/(HU_P_ − HU_U_)] × 100%, where HU, A, P, and U represent attenuation, arterial phase, portal phase, and unenhanced, respectively. The strong correlation (r = 0.91, *p* < 0.001) between hepatic perfusion and AEF was observed in 10 rabbits with VX2 liver tumor by Kim et al. (9). Thus, AEF is an ideal biomarker, which can be readily derived from routine triphasic liver CT examinations, to indirectly reflect the ratio of hepatic arterial perfusion to total perfusion (10). A recent study showed that elevated AEF of residual tumor after embolization was strongly associated with poor prognosis in drug-eluting beads (DEB) TACE-treated HCC patients (11). Thus, the purpose of the current study was to establish a prognostic model for progression in HCC patients after incomplete DEB-TACE by integrating AEF and clinical–radiological characteristics.

## 2 Materials and methods

The present analysis adhered to the Transparent Reporting of a Multivariable Prediction Model for Individual Prognosis or Diagnosis (TRIPOD) guideline (12). This retrospective study was approved by the institutional ethics committee and was conducted following the 1975 Declaration of Helsinki. The requirement for informed consent was waived, as all data were anonymized and collected from the electronic medical system.

### 2.1 Study population

**Figure 1:**
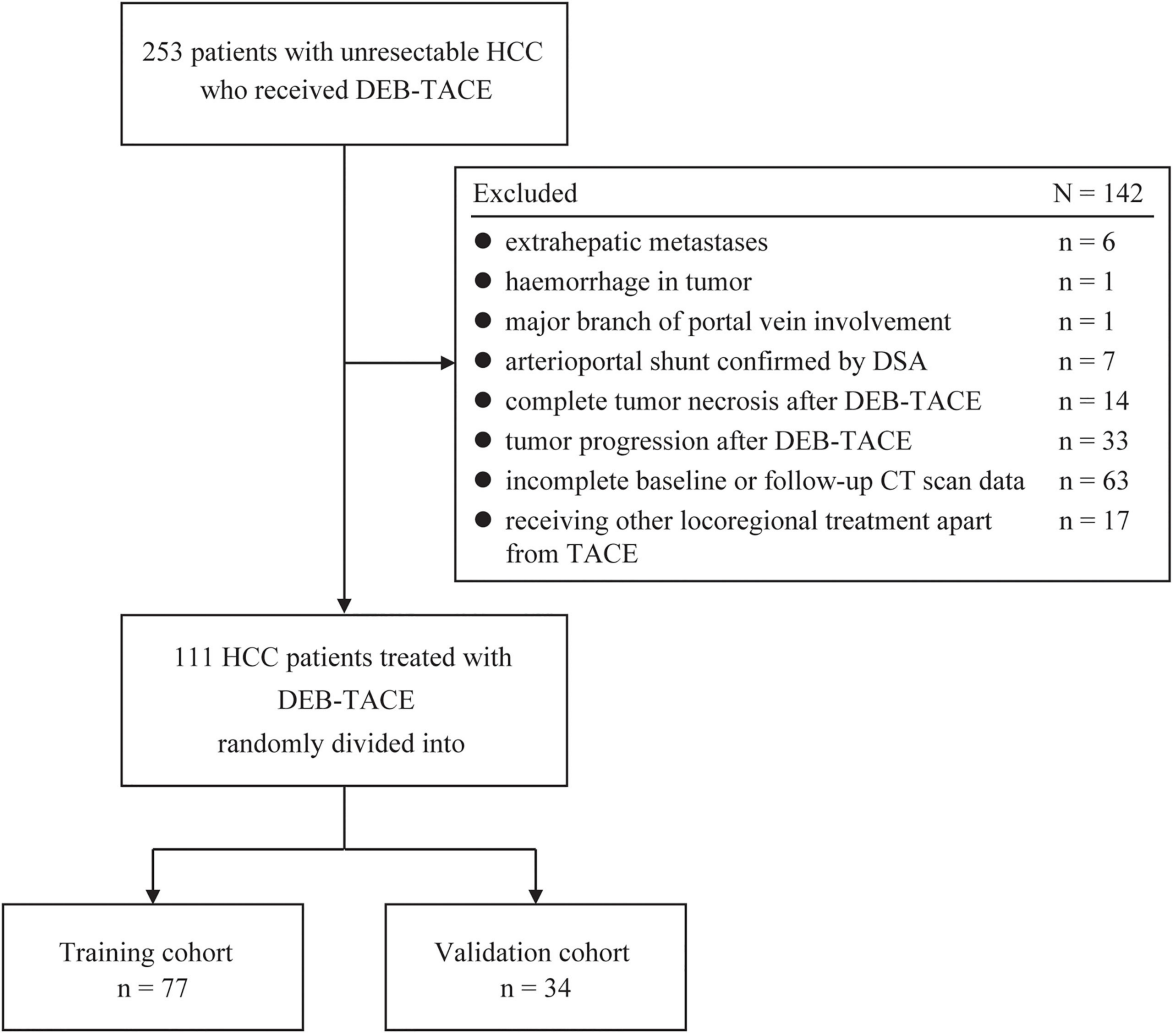
Flowchart showing the patient selection and grouping strategy for model derivation and validation. DEB-TACE, drug-eluting bead transarterial chemoembolization; HCC, hepatocellular carcinoma; DSA, digital subtraction angiography.

From October 2018 to March 2024, clinical and image information of 253 consecutive treatment-naive patients with unresectable HCC undergoing DEB-TACE were retrieved from the electronic medical database of our tertiary medical center. The follow-up was completed on 30 June 2024. Of these, 142 patients were excluded for the following reasons: (a) extrahepatic metastases (confounding factor for disease progression caused by the primary lesion, n = 6); (b) conditions not eligible for AEF measurement, comprising hemorrhage in HCC lesion (non-contrast agent caused high density, n = 1), HCC involving major branch of portal vein (low background enhancement caused by impaired portal venous blood supply, n = 1), arterioportal shunt (atypical lesion enhancement, n = 7), and complete tumor necrosis after first DEB-TACE treatment (n = 14); (c) missing triphasic CT scan data at baseline or at follow-up (n = 63); (d) disease progression after initial TACE treatment (n = 33); and (e) receiving any locoregional treatments other than TACE at follow-up (n = 17) (**Figure 1**). The diagnosis of HCC was either biopsy-proven or met the European Association for the Study of the Liver imaging criteria (13).

### 2.2 Procedure

DEB-TACE technique. All DEB-TACE procedures were performed by a team of interventional radiologists with over 10 years of experience. CalliSpheres (Jiangsu Hengrui Medicine Co. Ltd.) beads were loaded with 60 mg or 80 mg of epirubicin per vial and mixed with a non-ionic contrast medium to obtain the final injectable beads. The details of the DEB-TACE procedure has been described previously (11).

Follow-up protocol. The “on-demand” TACE procedure was scheduled at 6–12-week intervals based on CT or MR evidence of viable tumor or intrahepatic recurrence, unless contraindications were present. The median number of TACE sessions was 3 (range, 1–4), with one session in six patients, two in 39 patients, three in 51 patients, and four in 15 patients.

Image and AEF acquisition. CT acquisitions in this study were performed using three Siemens CT scanners: Somatom Definition, Somatom Definition AS, or Somatom Force (Siemens Healthcare, Erlangen, Germany). After unenhanced scanning, triphasic contrast-enhanced scans were performed following intravenous administration of 80–100 mL non-ionic contrast agent (Iopamidol, 370 mg I/mL, Bracco) at a rate of 2.5–3.0 mL/s through the antecubital vein using an automatic power injector. The arterial phase, portal venous phase, and equilibrium phase images were acquired at 30 s, 50 s, and 180 s after initiation of the contrast medium injection, respectively. The unenhanced, arterial phase, and portal venous phase datasets were then sent to a syngo.via workstation (Siemens Healthcare, Erlangen, Germany) for generating the quantitative color mapping of AEF by using the dedicated software in the MM Oncology mode. Typically, AEF was calculated based on the ratio of the absolute increment of attenuation during the arterial phase to the absolute increment of attenuation during the portal venous phase per pixel ( $\text{AEF=}\frac{{\text{HU}}_{\text{A}}{\text{-HU}}_{\text{U}}}{{\text{HU}}_{\text{P}}{\text{-HU}}_{\text{U}}}\times 100\%$ , where HU stand for attenuation, A for arterial phase, P for portal phase, and U for unenhanced). The resulting data are mapped to a spectral color scale that displays from black (0%) to red (100%) (10) (**Supplementary Figure S1**).

### 2.3 Outcome and predictors

Outcome. The outcome of interest was progression-free survival (PFS) (months), defined as the period between the TACE initiation and radiological detection of tumor progression (n = 109) or death (n = 2). Radiological progression was determined according to the modified response evaluation criteria in solid tumors (mRECIST) (14).

Clinical predictors. On the basis of the literature, the following clinical parameters were collected: sex, age, cause of HCC (hepatitis B infection or other disease), alanine transaminase (ALT), aspartate transaminase (AST), peripheric platelet count, albumin, total bilirubin, Child–Pugh classification, neutrophil-to-lymphocyte ratio (NLR), platelet−to−lymphocyte ratio (PLR), and alpha-fetoprotein. All the laboratory tests and physical examinations were performed within 3 days before the DEB-TACE procedure.

Radiological predictors. Radiological features of each patient were reviewed through pre-therapeutic liver CT examinations scheduled within 1 week before the DEB-TACE procedure. The first follow-up CT scan for treatment response assessment was conducted in 35.7 ± 4.1 days (range, 22–46) after initial treatment. Selected predictors of radiological characteristics included the diameter of the dominant HCC lesion (largest diameter of viable tumor on the axial section of arterial-phase images), margin appearance (smooth or non-smooth), enhancing capsule appearance in portal venous phase or equilibrium phase (absence or presence), lesion number (solitary or multifocal), tumor extent (unilobar or bilobar), and vascular invasion (presence of portal vein tumor thrombosis). The dominant tumor was determined as the largest measurable target lesion per patient. All image analyses, incorporating dominant lesion determination, radiological characteristics identification, and treatment response assessment, were conducted by two nonauthor abdominal radiologists (14 years and 10 years of experience in abdominal radiology) who were unfamiliar with the study design, and any discrepancy during analysis was resolved by consensus.

AEF of residual tumor. The AEF of residual tumor (hereafter, AEF-RT) was measured in the AEF color map derived from the first follow-up CT scan. The measuring procedure was consistent with the previous study (11). In short, two authors (B.C. and D.Q.X.), blinded to patient outcomes, manually delineated regions of interest on three transverse planes. The mean AEF-RT, calculated across all three planes, was used for analysis.

### 2.4 Statistical analysis

Continuous variables with normal distribution are expressed as the mean ± standard deviation, and those with non-normal distribution are expressed as the median (interquartile range). Categorical variables are expressed as frequencies (percentages). Missing data in predictors were assumed to be random, and five imputations using chained equations were performed to correct for bias. Unless otherwise specified, statistical analyses were performed using GraphPad Prism (Version 8.3.4) and packages of “mice,” “survival,” “survminer,” “rms,” and “compareC” in R software (Version 4.4.1) (http://cran.r-project.org).

Model building procedure. The Least Absolute Shrinkage and Selection Operator (LASSO) regression was applied to select the most significant predictors for PFS from the 19 candidate predictors mentioned above in the training cohort. Tenfold cross-validation of LASSO regression was performed using the R package “glmnet.” The final predictors were determined by a stepwise Cox proportional hazards regression algorithm from all significant predictors selected by the LASSO regression at the lambda value corresponding to the minimum error. The proportional hazards assumption of the models was tested by examining the plots of scaled Schoenfeld residuals against time for each variable in the final model.

Generating different progression risk categories. To generate different risk categories, the weighted sum of regression coefficients from the Cox model, or the linear predictor, was computed. Survival curves according to the risk categories were plotted using Kaplan–Meier (KM) method, allowing a visual comparison of discrimination. The more widely separated are the curves, the better is the discrimination. The median PFS of each risk category, *p*-values of the log-rank test, hazard ratio (HR), and *p*-values of the Wald coefficient test were also reported. The calibration, or prediction accuracy of PFS, in the different risk groups was assessed graphically following the procedure proposed by Royston and Altman (15). Briefly, the model-predicted mean survival curves were created by applying fractional polynomial regression to approximate the log baseline cumulative hazard function as a smooth function of time. Then, the KM estimate and model-predicted survival curves were superimposed on the same plot to examine the model calibration visually.

Model performance assessment. Harrell’s concordance index (C-index, where 0.5 indicates non-informative discrimination and 1 represents perfect discrimination) was computed to assess the model performance of discrimination. The plots of the time-dependent concordance index were also created to visualize the discrepancy of the C-index between models at each time point (months).

The calibration performance was assessed by the Brier score (from 0 to 1, where 0 indicates better calibration) at 6 months and 12 months using the R package “riskRegression.” Additionally, integrated Brier score (IBS) was calculated with inverse probability of censoring weights to adjust for right-censored data using the R package “SurvMetrics” (16).

## 3 Results

### 3.1 Clinical characteristics and PFS

In total, 111 patients were included in the study, with 77 and 34 randomly allocated to the training and validation cohort, respectively. **Table 1** reports the clinical and disease related information. The median follow-up time was 25 months for the whole cohort, 24 months for the training cohort, and 25 months for the validation cohort. Progression was recorded in 91 patients, with 66 in training cohort and 25 in the validation cohorts. The median PFS was 7 months for both the whole and training cohorts and 8 months for validation cohort.

**Table 1:** Clinical and disease-related information of 111 patients with HCC.

Characteristics	Whole cohort (n = 111)	Training cohort (n = 77)	Validation cohort (n = 34)	*p*
Median age (years)	57 (24, 84)^†^	60 (24, 84)^†^	52.5 (34, 81)^†^	0.051
Sex (male/female)	97 (87.4%)/14 (12.6%)	67 (87.0%)/10 (13.0%)	30 (88.2%)/4 (11.8%)	0.896
Cause of HCC (Hepatitis B/Other)	97 (87.4%)/14 (12.6%)	68 (88.3%)/9 (11.70%)	29 (85.3%)/5 (14.7%)	0.758
ALT (U/L)^‡^	42.0 (28, 47)	41.0 (26.0, 46.8)	45.4 (30.5, 50.0)	0.324
AST (U/L)^‡^	53.0 (36, 69)	51.0 (36.0, 69.0)	55.0 (37.8, 69.0)	0.797
NLR^‡^	2.6 (2.0, 4.0)	2.5 (2.0, 4.0)	2.9 (1.9, 4.3)	0.537
PLR^‡^	133.0 (100.0, 184.4)	133.0 (108.2, 187.8)	110.4 (84.4, 165.8)	0.089
PLT (×10^9^/L)^‡^	165.0 (111.0, 199.0)	166.2 (111.5, 189.5)	151.5 (100.3, 236.8)	0.945
ALB (g/L)^‡^	36.6 (34.8, 39.0)	36.6 (34.7, 39.1)	36.8 (34.8, 39.2)	0.790
TBIL (μmol/L)^‡^	17.6 (13.9, 20.3)	17.6 (14.0, 20.9)	17.7 (13.0, 19.5)	0.936
Child-Pugh class (A/B)	91 (82.0%)/20 (18.0%)	65 (84.4%)/12 (15.6%)	26 (76.5%)/8 (23.5%)	0.315
AFP (ng/mL) (≤400/>400)	63 (56.8%)/48 (43.2%)	40 (51.9%)/37 (48.1%)	23 (67.7%)/11 (32.3%)	0.124
No. of lesions (solitary/multifocal)	56 (50.5%)/55 (49.5%)	39 (50.7%)/38 (49.3%)	17 (50.0%)/17 (50.0%)	0.950
Diameter (cm)	9.2 ± 4.0	9.4 ± 4.2	8.8 ± 3.5	0.507
Vascular invasion (absent/present)	76 (68.5%)/35 (31.5%)	51 (66.2%)/26 (33.8%)	25 (73.5%)/9 (26.5%)	0.446
Tumor extent (unilobar/bilobar)	79 (71.2%)/32 (28.8%)	53 (68.8%)/24 (31.2%)	26 (76.5%)/8 (23.5%)	0.413
Enhancing capsule (absent/present)	63 (56.8%)/48 (43.2%)	43 (55.8%)/34 (44.2%)	20 (58.8%)/14 (41.2%)	0.770
Margin appearance (smooth/non-smooth)	62 (55.9%)/49 (44.1%)	43 (55.8%)/34 (44.2%)	19 (55.9%)/15 (44.1%)	0.997
AEF of residual tumor (%)	44.3 ± 15.9	43.8 ± 15.9	45.7 ± 16.0	0.558
Median PFS (months)	7 (6, 10)^§^	7 (6, 9)^§^	8 (5, 17)^§^	–

Unless otherwise indicated, data in parentheses are percentages. AEF, arterial enhancement fraction; AFP, α-fetoprotein; ALT, alanine transaminase; AST, aspartate transaminase; HCC, hepatocellular carcinoma; NLR, neutrophil-to-lymphocyte ratio; PFS, progression-free survival; PLT, peripheric platelet count; PLR, platelet-to-lymphocyte ratio; PR, partial response; SD, stable disease; TBIL, total bilirubin.

^†^Data in parentheses are range.

^‡^Data are expressed as median (interquartile range).

^§^Data in parentheses are 95% confidence interval.

### 3.2 Construction of multivariate Cox proportional hazards model for PFS

**Figure 2:**
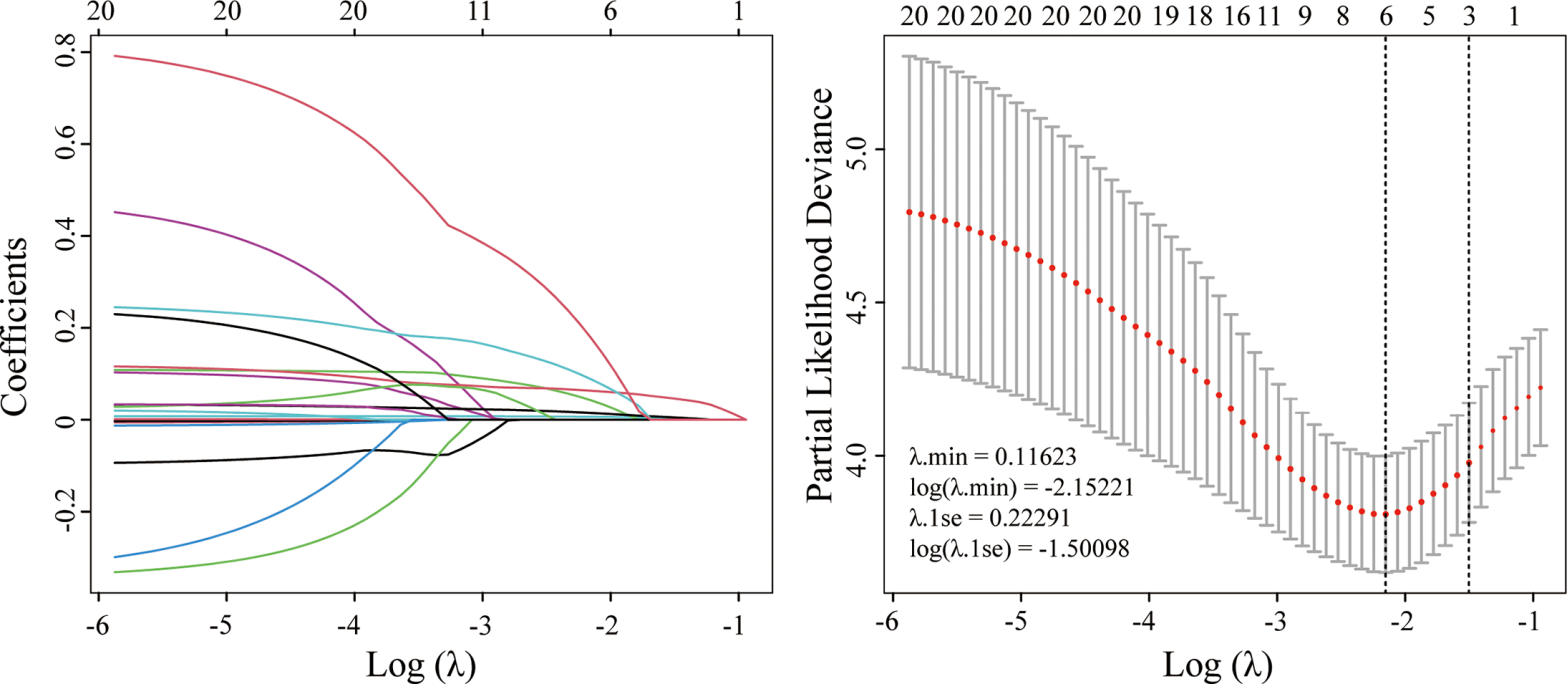
LASSO regression for candidate predictors selection.

**Figure 3:**
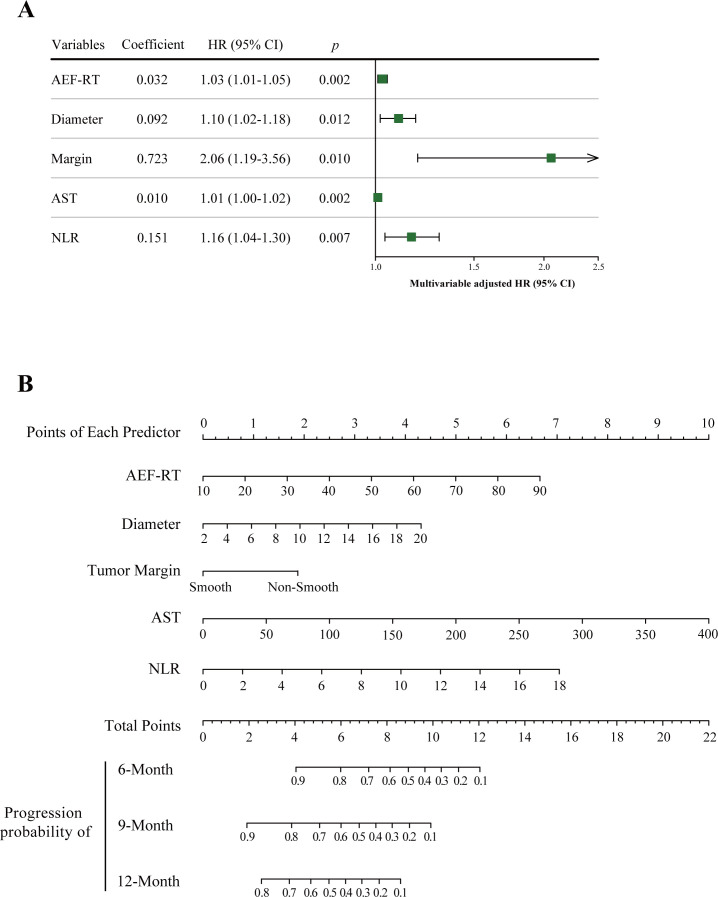
Visualization of the ADMAN model parameters **(A)** and ADMAN model-based nomogram **(B)**. Instruction for ADMAN model-based nomogram: Locate an individual patient’s value on each independent variable axis, and then draw a line upward to obtain the points for each variable. Next, locate the sum of these points on the total points axis, and draw a line downward to the progression axis to obtain the probability of 6-, 9-, and 12-month PFS. AEF-RT = arterial enhancement fraction of residual tumor, AST, aspartate transaminase; DEB-TACE, drug-eluting beads transarterial chemoembolization; HCC, hepatocellular carcinoma; NLR, neutrophil-to-lymphocyte ratio; PFS, progression-free survival.

In this study, the optimal λ value was chosen as 0.11623 (λ.min), leading to the selection of six variables with nonzero coefficients using LASSO regression (**Figure 2**, **Table 2**), five of which were retained as significant predictors in the final Cox model. These variables included AEF-RT, tumor diameter, margin appearance (smooth or non-smooth), AST, NLR, and all treated as continuous variables except margin appearance. The parameters of the final Cox model, referred to as the ADMAN model, are depicted in **Figure 3A**. A nomogram based on the ADMAN model was constructed to estimate the probability of PFS at 6 months, 9 months, and 12 months after TACE for individual patients (**Figure 3B**).

**Table 2:** Features entered the LASSO for PFS in the training cohort.

Variables	Coefficients (λ.min)	Coefficients (λ.1se)
Age (y)	–	–
Sex (male/female)	–	–
Cause of HCC (hepatitis B/other)	–	–
ALT (U/L)	–	–
AST (U/L)	0.006412359	0.003018912
NLR	0.042218270	–
PLR	–	–
PLT (×10^9^/L)	–	–
ALB (g/L)	–	–
TBIL (μmol/L)	–	–
Child-Pugh class (A/B)	–	–
AFP (ng/mL) (≤400/>400)	–	–
No. of lesions (solitary/multifocal)	0.099686063	–
Diameter (cm)	0.063100231	0.046008561
Vascular invasion (absent/present)	–	–
Tumor extent (unilobar/bilobar)	–	–
Enhancing capsule (absent/present)	–	–
Margin appearance (smooth/non-smooth)	0.177587833	–
AEF of residual tumor (%)	0.014771557	0.004969961

The “–” means zero coefficient of the variable at respective λ value. AEF-RT, arterial enhancement fraction of residual tumor; AFP, α-fetoprotein; ALT, alanine transaminase; AST, aspartate transaminase; HR, hazard ratio; LASSO, least absolute shrinkage and selection operator; NLR, neutrophil-to-lymphocyte ratio; PFS, progression-free survival; PLR, platelet−to−lymphocyte ratio; PLT, peripheric platelet count; TBIL, total bilirubin.

A nested DMAN model was developed by deleting AEF-RT from the complete model to determine the contribution of AEF-RT in predicting tumor progression. For the model performance, the C-index of the DMAN model was marginally significantly lower than that of the ADMAN model (0.706 vs. 0.754, *p* = 0.051). However, as comparing C-indexes of two nested models is a low-power procedure, the likelihood ratio χ^2^ test was used instead to compare the ADMAN and DMAN models (17). A significant loss of predictive power was observed in the likelihood ratio test when AEF-RT was removed from the ADMAN model (χ^2^ = 9.685, *p* = 0.002).

### 3.3 ADMAN-based risk categories

The ADMAN linear predictor for observation in the training cohort was computed based on the following formula:


$$\begin{array}{c} \text{ADMAN linear predictor}=0.032\times \text{AEF }\left(\text{value without percent sign}\right)\\ +\;0.092\;\times \text{ Diameter of dominant tumor }\left(\text{cm}\right)\\ +\;0.723\;\times \text{ Margin appearance }\left(0=\text{smooth},\;1=\text{non‐smooth}\right)\\ +\;0.010\;\times \text{ AST }\left(\text{U}/\text{L}\right)\\ +\;0.199\;\times \text{ NLR} \end{array}$$

The median ADMAN linear predictor (3.55) was used as the cutoff to categorize patients into high- and low-risk groups. This cutoff was then applied to the validation cohort for subsequent grouping.

**Figure 4:**
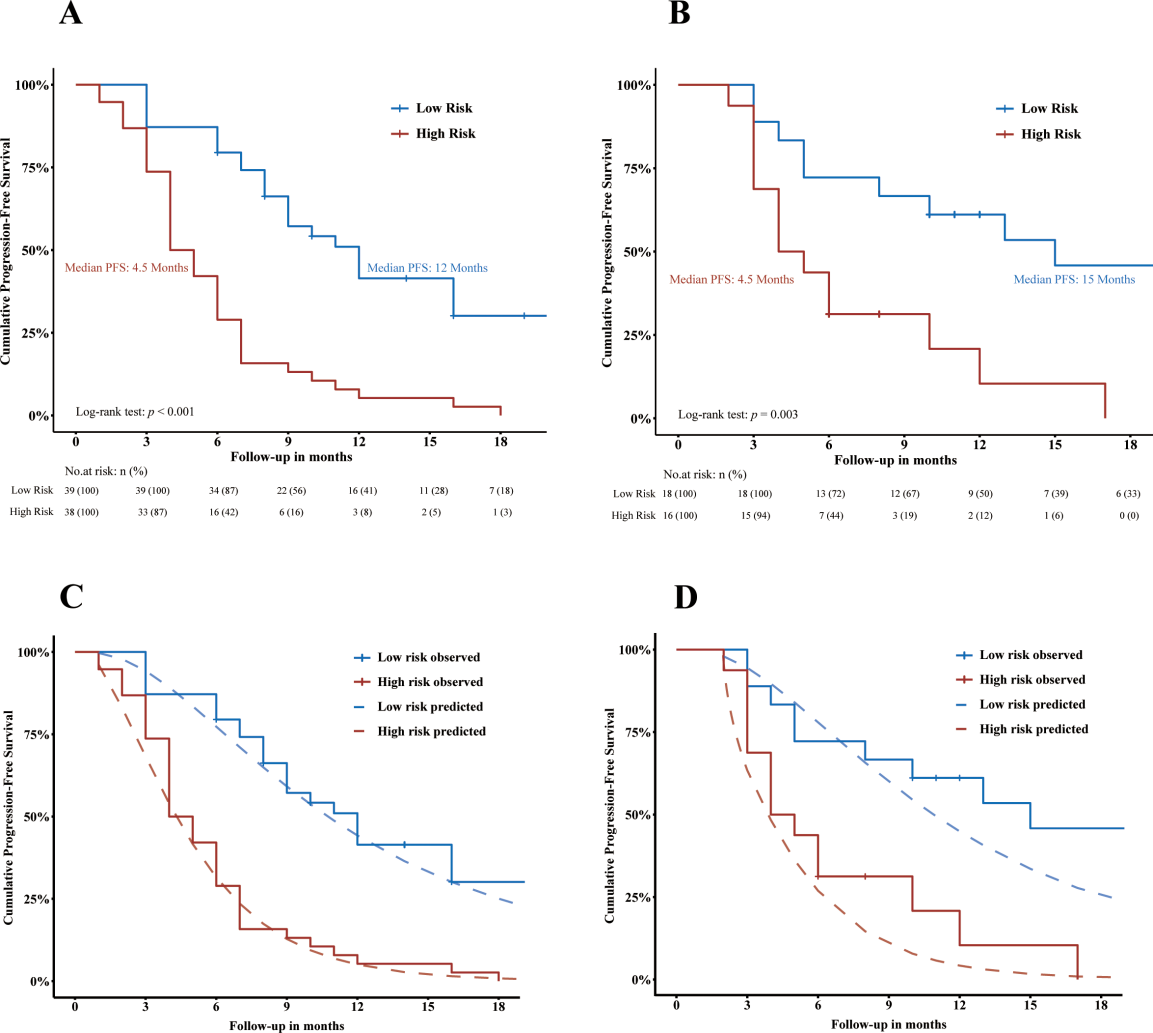
Visualization of the discrimination **(A, B)** and calibration **(C, D)** of ADMAN model. Kaplan–Meier curves showing the PFS of HCC patients stratified by progression risk in training cohort **(A)** and in validation cohort **(B)**. Plots depicting Kaplan–Meier estimate survival curves (jagged line) against ADMAN model-predicted mean survival curves (smooth dash line) in the training cohort **(C)** and validation cohort **(D)**. PFS, progression-free survival.

In the training cohort, good separation of survival curves was achieved for the high- and low-risk groups according to ADMAN-based risk categories [median PFS: 4.5 months vs. 12 months, *p* < 0.001; HR = 4.69 (95% CI, 2.68–8.19), *p* < 0.001] (**Figure 4A**). In the validation cohort, there remained a significant difference in PFS between the high- and low-risk groups [median PFS: 4.5 months vs. 15 months, *p* = 0.003; HR = 3.52 (95% CI, 1.47–8.43), *p* = 0.005) (**Figure 4B**).

The calibration of the ADMAN-based risk categorization was visualized in **Figures 4C and D**. Good agreement was observed between the KM estimate and the ADMAN-predicted survival curves in the training cohort (**Figure 4C**) and was maintained in the validation cohort (**Figure 4D**).

### 3.4 Comparison of PFS between models

Given the lack of an established strategy for predicting PFS in HCC treated with TACE, we compared the performance of the ADMAN model with other widely recognized prognostic scoring systems designed for overall survival prediction, including the hepatoma arterial-embolization prognostic (HAP) score (18), modified HAP score (3), mHAP-II score (19), Six-and-Twelve score (20), and Up-to-11 criteria (21). The scores assignment and classification strategies are detailed in **Supplementary Table S1**. To avoid the training bias, the comparison was also performed in the validation set.

**Figure 5:**
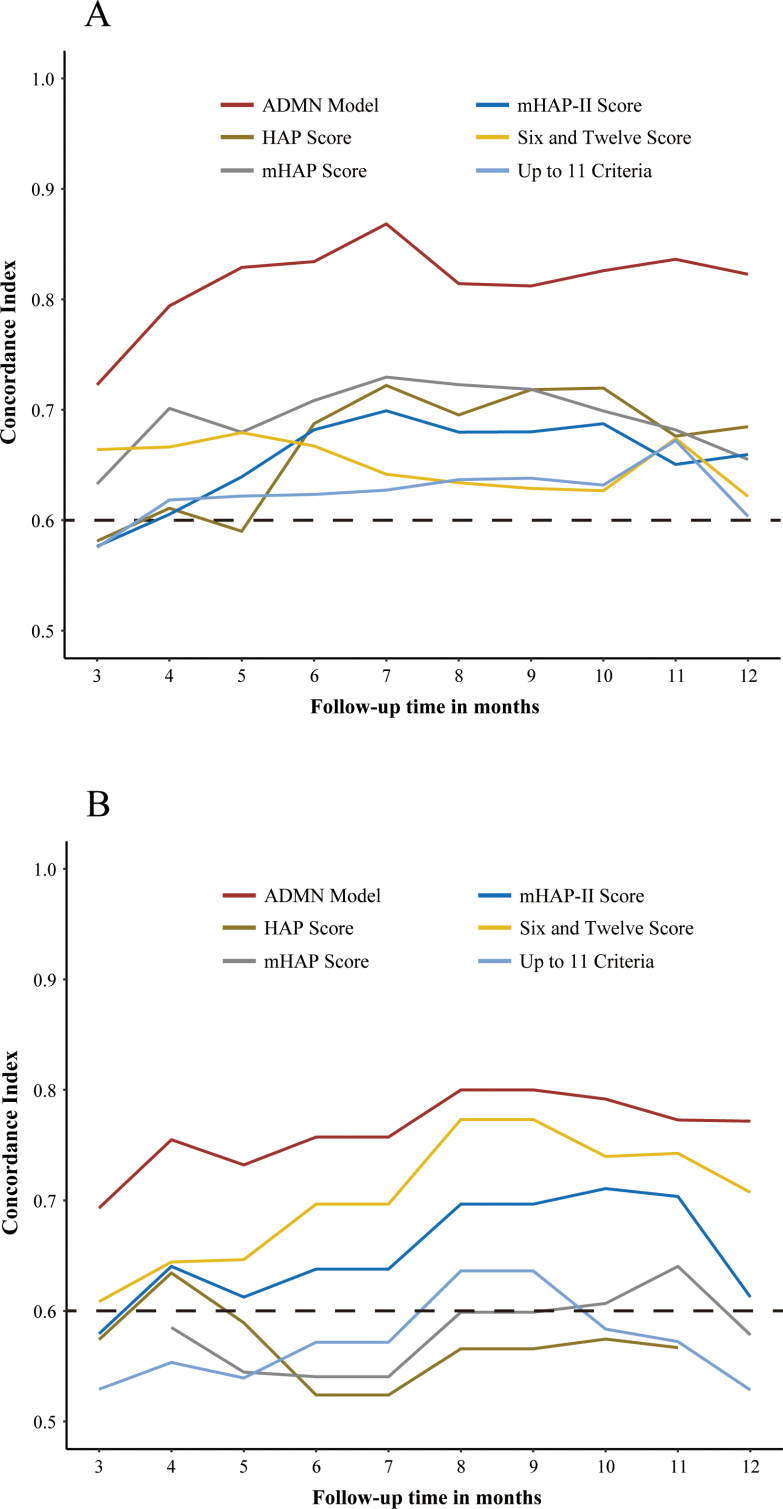
Plots of time-dependent C-index showing the C-index of the ADMAN model and five other prognostic models for PFS over time in the training **(A)** and validation cohorts **(B)**.

In the training cohort, the ADMAN model (0.75) demonstrated a significantly higher C-index than the other five prognostic score systems (*p <* 0.05 for all) (**Table 3**). In the validation cohort, the ADMAN model maintained sufficient discriminatory performance, with a significantly higher C-index (0.71) compared to the HAP Score (0.55, *p* = 0.041), mHAP Score (0.54, *p* = 0.033), and the Up-to-11 criteria (0.53, *p* = 0.004) while demonstrating a marginally higher C-index than the mHAP-II Score (0.61, *p* = 0.080) and Six-and-Twelve Score (0.64, *p* = 0.231) (**Table 3**). Additionally, the plots of time-dependent C-index were created to visualize the differences in the performance of discrimination between models over time. The ADMAN model demonstrated the consistently highest C-index during follow-up either in the training (**Figure 5A**) or validation cohort (**Figure 5B**).

**Table 3:** C-index of different models for PFS in the training and validation cohorts.

Models	Training cohort	*p* ^†^	Validation cohort	*p* ^†^
ADMAN Model	0.75 (0.69–0.82)	–	0.71 (0.60–0.83)	–
HAP Score	0.62 (0.55–0.71)	**0.004**	0.55 (0.47–0.70)	**0.041**
mHAP Score	0.65 (0.58–0.73)	**0.014**	0.54 (0.40–0.69)	**0.033**
mHAP-II Score	0.63 (0.55–0.70)	**0.002**	0.61 (0.48–0.74)	0.080
Six-and-Twelve Score	0.63 (0.56–0.70)	**<0.001**	0.64 (0.53–0.75)	0.231
Up-to-11 criteria	0.60 (0.53–0.67)	**<0.001**	0.53 (0.44–0.65)	**0.004**

HAP, hepatoma arterial-embolization prognostic score; mHAP, modified hepatoma arterial-embolization prognostic score; PFS, progression-free survival.

^†^ *p*-value for comparing the C-index of ADMAN model and other prognostic model.

Bold values represent p-value < 0.05.

The calibration of each model was assessed by Brier score of 6 months and 12 months PFS. In the training cohort, the Brier score of the ADMAN model was significantly lower than those of the other existing models, indicating better calibration (**Table 4**). In the validation cohort, the ADMAN model remained to have significantly lower 6-month Brier score than HAP Score, mHAP Score, mHAP-II Score, and Up-to-11 criteria, while it had significantly lower Brier score than mHAP Score and Up-to-11 criteria at 12 months (**Table 4**). The integrated Brier score (IBS) extends the Brier score over time, evaluating the overall performance of probability forecasts across multiple time points. It is calculated by integrating the Brier score over time, offering a comprehensive view of a model’s performance in time-series forecasting. In the training cohort, the ADMAN model (0.086) had a lower IBS than HAP Score (0.100), mHAP Score (0.099), mHAP-II Score (0.098), Six-and-Twelve Score (0.103), and Up-to-11 criteria (0.104). In the validation cohort, the IBS of the ADMAN model showed moderate calibration (0.170), while the IBS of other models indicated poor calibration [HAP Score (0.285), mHAP Score (0.291), mHAP-II Score (0.261), Six-and-Twelve Score (0.246), Up-to-11 criteria (0.358)].

**Table 4:** Brier score of different models for PFS at 6 months and 12 months.

Time point	Training cohort	*p* ^†^	Validation cohort	*p* ^†^
Models				
6-Month				
ADMAN	0.161	–	0.183	
HAP Score	0.219	**0.009**	0.247	**0.041**
mHAP Score	0.210	**0.025**	0.257	**0.012**
mHAP-II Score	0.220	**0.004**	0.232	**0.049**
Six-and-Twelve Score	0.225	**<0.001**	0.214	0.238
Up-to-11 criteria	0.233	**<0.001**	0.244	**0.047**
12-Month				
ADMAN	0.140	–	0.191	–
HAP Score	0.203	**0.007**	0.248	0.054
mHAP Score	0.202	**0.014**	0.240	**0.049**
mHAP-II Score	0.209	**0.006**	0.234	0.158
Six-and-Twelve Score	0.211	**0.001**	0.196	0.888
Up-to-11 criteria	0.215	**<0.001**	0.243	**0.041**

HAP, hepatoma arterial-embolization prognostic score; mHAP, modified hepatoma arterial-embolization prognostic score; PFS, progression-free survival.

^†^ *p*-value for comparing the brier score of ADMAN model and other prognostic model.

Bold values represent p-value < 0.05.

## 4 Discussion

Existing scoring systems for TACE commonly incorporate pre-treatment characteristics, serum tumor markers, liver function tests, and tumor burden as prognostic indicators (1, 3, 18–21). However, despite these systems, TACE remains the preferred palliative treatment for patients ineligible for surgery due to the absence of a widely accepted pre-treatment scoring system, as noted in various international guidelines (1, 22, 23). Unfortunately, 70%–80% of patients would eventually die due to tumor progression following repeat TACE procedures (2). This highlights the importance of transitioning from TACE to systemic therapies before liver function is compromised by repeated and ineffective treatments. Our purpose in developing this post-treatment scoring system is grounded in clinical practice, aiming to predict the suitability of TACE for patients based on certain post-treatment disease characteristics, such as the blood flow perfusion status of residual lesions.

In the present study, we integrated a perfusion-like parameter of residual tumor with other clinical and radiological predictors to preliminarily develop and validate a prognostic model for PFS in patients undergoing DEB-TACE. The ADMAN model allows for the categorization of patients into distinct prognostic risk groups, enabling tailored follow-up schedules or alternative treatments for high-risk individuals. A comparison of five existing prognostic scores demonstrated that the ADMAN model exhibited promising performance in predicting PFS, which was further confirmed in the validation cohort. However, slightly declined performance of the ADMAN model in the validation cohort warrants further discussion. In the validation cohort, C-index of the ADMAN model consistently outperformed that of the HAP Score (*p* = 0.041), mHAP Score (*p* = 0.033), and Up-to-11 criteria (*p* = 0.004). These statistically significant advantages may be attributable to differences in the modeling cohorts, as each of these three models was developed in Western populations (3, 18, 21), where the etiological factors for HCC, such as hepatitis C virus, alcohol, obesity, and metabolic syndrome, differ notably from those in Asian populations, where hepatitis B virus predominates. In contrast, the mHAP-II score, developed in a Korean population, aligns more closely with the etiological and demographic characteristics of Chinese patients (19). As a result, the ADMAN model’s C-index was only slightly higher than the mHAP-II score. The Six-and-Twelve score, developed in an entirely Chinese cohort, demonstrated the most comparable performance to the ADMAN model (20). Regarding the statistically insignificant Brier score of the ADMAN model, we hypothesize that this result may stem not only from demographic factors but also from the increasing proportion of censored observations over time, which impacts the dispersion of the empirical Brier score. In our cohort, the median PFS was 7 months [95% CI (6, 10)], leading to higher Brier score dispersion at time points with concentrated censoring. This may explain why the ADMAN model’s Brier score showed no significant difference from that of other models in the validation cohort. To address this limitation, we included the IBS, which evaluates prediction inaccuracy over an interval rather than at a single time point by integrating loss functions. The IBS provides a more comprehensive measure of model performance over time and further supports the robustness of the ADMAN model.

Furthermore, the applicability of the ADMAN model being limited to patients with incomplete embolization may be considered a limitation. However, from our perspective, patients achieving complete response after initial TACE tend to maintain a relatively stable condition, whereas those with incomplete embolization are more likely to experience disease progression in the short term, despite repeated TACE attempts. Previous studies have also identified complete response as a significant protective factor for survival (24).The underlying mechanism may be attributed to intratumoral hypoxia induced by incomplete embolization. Hypoxia within the tumor microenvironment has been shown to promote epithelial–mesenchymal transition in cancer cells, thereby enhancing tumor aggressiveness and resistance to treatment (25, 26). Moreover, hypoxia triggers neo-angiogenesis and vascularization, processes that can be reflected by the AEF of tumor tissue (9).

Unlike chemoembolization, radioembolization delivers radioactive microspheres directly to tumors via super-selective catheterization, providing localized radiation to kill tumor cells without significant embolic effects. Although this technique has minimal impact on arterial blood flow, its potential influence on tumor angiogenesis or perfusion remains uncertain. A recent study suggests that hypoperfused primary liver tumors treated with Y-90 may have worse clinical outcomes compared to hyperperfused tumors (27). In that study, tumor perfusion was visually assessed by researchers using preoperative CT or MRI. Hypoperfused lesions were defined as those with less enhancement than the surrounding liver parenchyma, while hyperperfused lesions exhibited similar or greater enhancement. This raises an intriguing question: could AEF assessment serve as a complementary method to visual evaluation? Given that AEF can be easily integrated into routine liver CT scans with less than 2 min of additional postprocessing time, future TARE studies could explore its feasibility for preoperative perfusion evaluation and for assessing changes in postoperative blood flow.

Although not directly applied in the current study, artificial intelligence (AI)-based techniques hold significant potential for overcoming limitations in AEF-based lesion analysis. AI-driven methods can provide a more comprehensive assessment of lesion perfusion dynamics, addressing challenges such as tumor heterogeneity, post-embolization alterations in blood supply, and high-perfusion artifacts caused by inflammation in necrotic region. For instance, stochastic resonance, as demonstrated by Dakua et al. in aneurysm segmentation, enhances contrast in low-signal environments, improving lesion-background differentiation and offering potential applications in AEF analysis (28, 29). Similarly, multi-modality registration methods, such as diffeomorphic mapping, ensure consistent segmentation across imaging protocols. Level-set methods integrated with denoising techniques, including maximal overlap discrete wavelet transforms, further refine lesion contours in noisy datasets (30, 31). Advanced preprocessing and regularization techniques further improve model robustness. For example, Dense-PSP-UNet employs contrast limited adaptive histogram equalization (CLAHE) to enhance boundary visualization and reduce noise in low-contrast images, preserving critical features and improving segmentation accuracy (32). Res-PAC-UNet incorporates Pyramid Atrous Convolution modules to capture multi-scale features, enriching contextual information and resolving ambiguous boundaries (33). Additionally, synthetic oversampling methods like SMOTE address class imbalance by generating synthetic samples for underrepresented categories, thereby improving model generalization and representation (34). Collectively, these techniques—contrast enhancement, multi-scale feature extraction, and data balancing—help mitigate overfitting and improve the generalizability of AEF-based models, establishing a foundation for robust, accurate predictive tools in complex imaging environments.

Apart from the inherent limitations of its retrospective design, our study has several other constraints. First, the relatively small sample size, especially in the validation cohort, limits the generalizability of our findings to broader patient populations. External validation with larger, independent cohorts is necessary to confirm the robustness of the ADMAN model in different clinical settings. Second, this study excluded patients who underwent chemoembolization with Lipiodol due to high-density artifacts caused by iodized oil deposits. These artifacts significantly hinder the identification of residual tumors and distort the AEF color map, making it unsuitable for evaluating postoperative imaging in these patients. This exclusion restricts the applicability of our findings to this subgroup of patients. Third, the model was developed and validated within an Asian population predominantly affected by hepatitis B virus, which limits its applicability to populations with different etiological profiles, such as those characterized by hepatitis C virus, alcohol-related liver disease, or metabolic syndrome. Variations in underlying etiologies may influence the performance and generalizability of the ADMAN model. Lastly, technical limitations in AEF measurement remain a challenge. Post-embolization changes, such as necrotic inflammation and altered blood perfusion, can introduce variability in measurements. Additionally, manual delineation of residual tumors on the AEF color map may lead to interobserver variability. These issues might be mitigated by integrating AI-based lesion segmentation techniques, which could standardize measurements and enhance the consistency and reliability of the results.

In conclusion, this pilot study underscores the potential utility of AEF-RT in predicting progression after DEB-TACE. The ADMAN model enabled the progression risk stratification and individualized estimation of PFS in patients with HCC undergoing DEB-TACE. Further external validation in independent cohorts with larger sample sizes is necessary to confirm the robustness of the ADMAN model.

## Data Availability

The raw data supporting the conclusions of this article will be made available by the authors, without undue reservation.
