# Supplementary material for: Pilot study of an arterial enhancement fraction-based model for progression prediction in HCC undergoing chemoembolization
Source: Front Oncol. 2025 Feb 19;15:1489450. doi: 10.3389/fonc.2025.1489450 (PMC11879821; doi:10.3389/fonc.2025.1489450)
Supplement: Supplementary file 1 [file DataSheet1.docx]

# Supplementary Material

| **Supplementary Table 1.** The scores assignment and classification strategies of existing prognostic systems | | |
| --- | --- | --- |
| **Prognostic Score Systems** | **Scores Assignment** | **Classification Strategies** |
| HAP | (1 point for each item, 0 if did not fit) ·Alb < 36 g/dl ·AFP > 400 ng/ml ·TBIL > 17 μmol/L ·Tumor size > 7 cm | A: 0 B: 1 C: 2 D: > 2 |
| mHAP | (1 point for each item, 0 if did not fit) ·Alb < 36 g/dl ·AFP > 400 ng/ml ·Tumor size > 7 cm | A: 0 B: 1 C: 2 D: > 2 |
| mHAP-II | (1 point for each item, 0 if did not fit) ·Alb < 36 g/dl ·AFP > 400 ng/ml ·TBIL > 17 μmol/L ·Tumor size > 7 cm ·Tumor number >= 2 | A: 0 B: 1 C: 2 D: > 2 |
| Six-and-Twelve | Tumor size + Tumor number | Sum no more than 6 Sum between 6 and 12 Sum beyond 12 |
| Up-to-11 | Tumor size + Tumor number | Sum no more than 11 Sum beyond 11 |
| Alb = albumin, AFP = α-fetoprotein, TBIL = total bilirubin | | |

**Supplementary Figure 1**― The quantitative color mapping of AEF (**d**) for AEF-RT measuring was generated from unenhanced (**a**), arterial phase (**b**), portal venous phase (**c**) images after DEB-TACE.


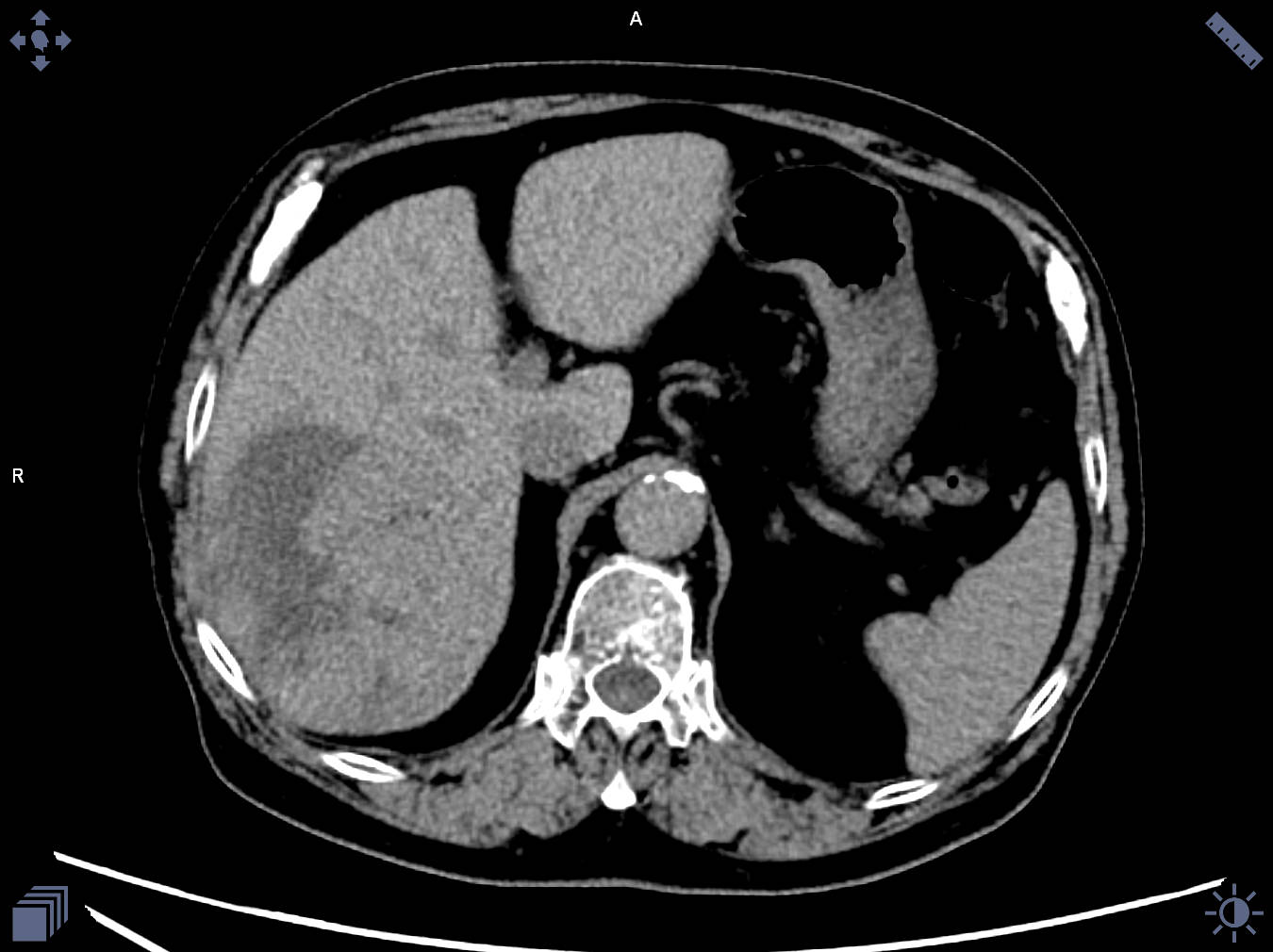

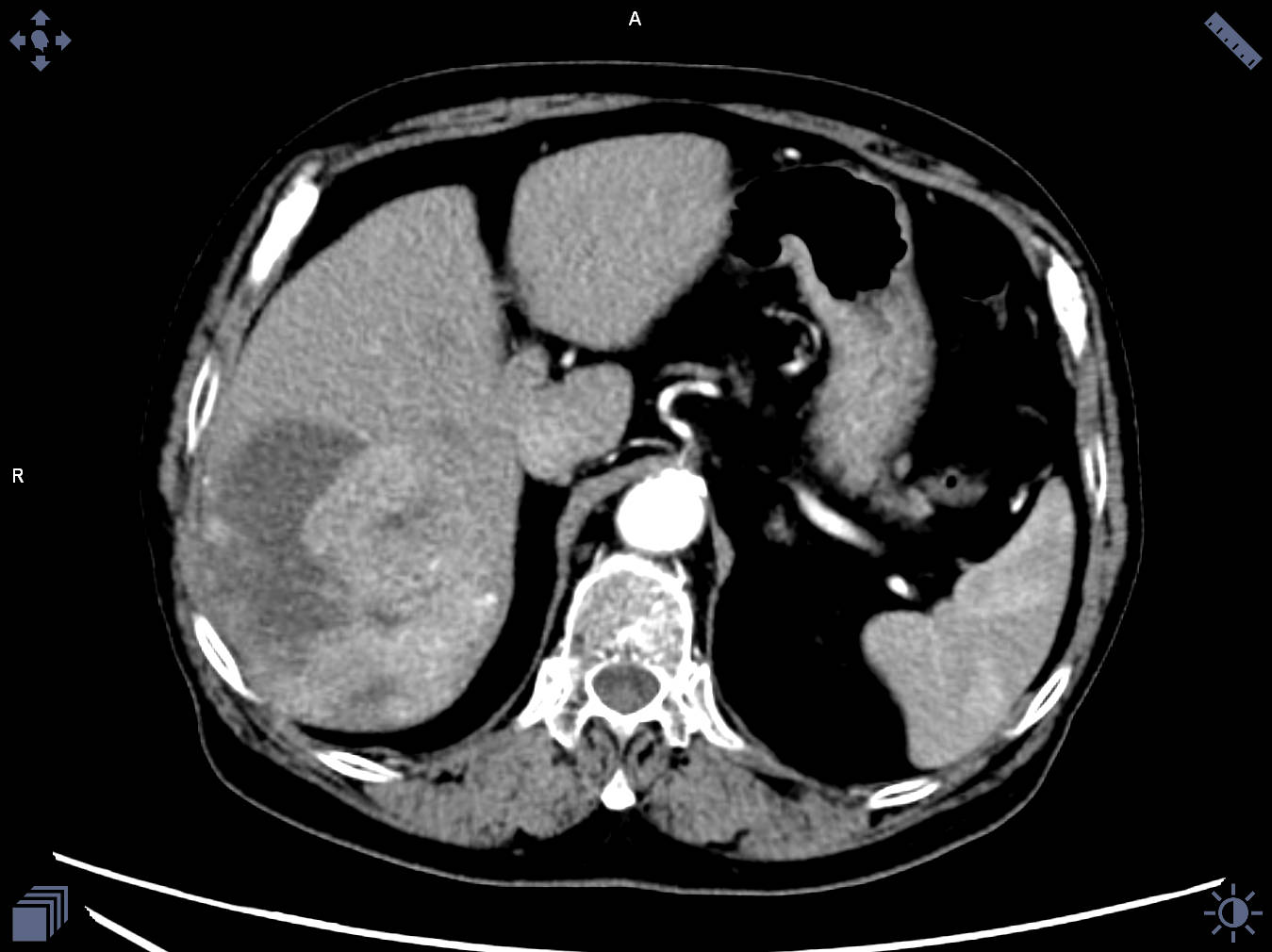

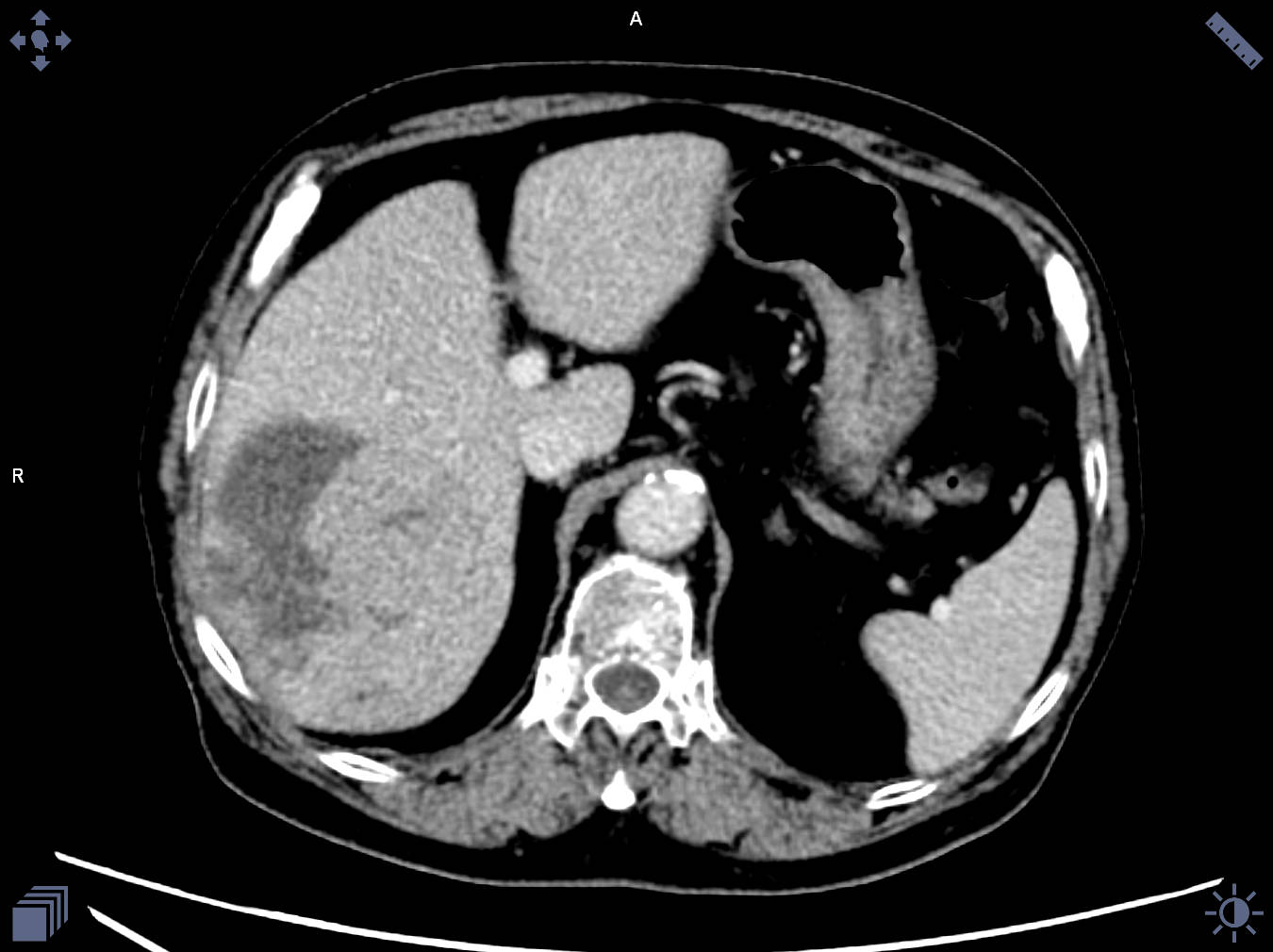

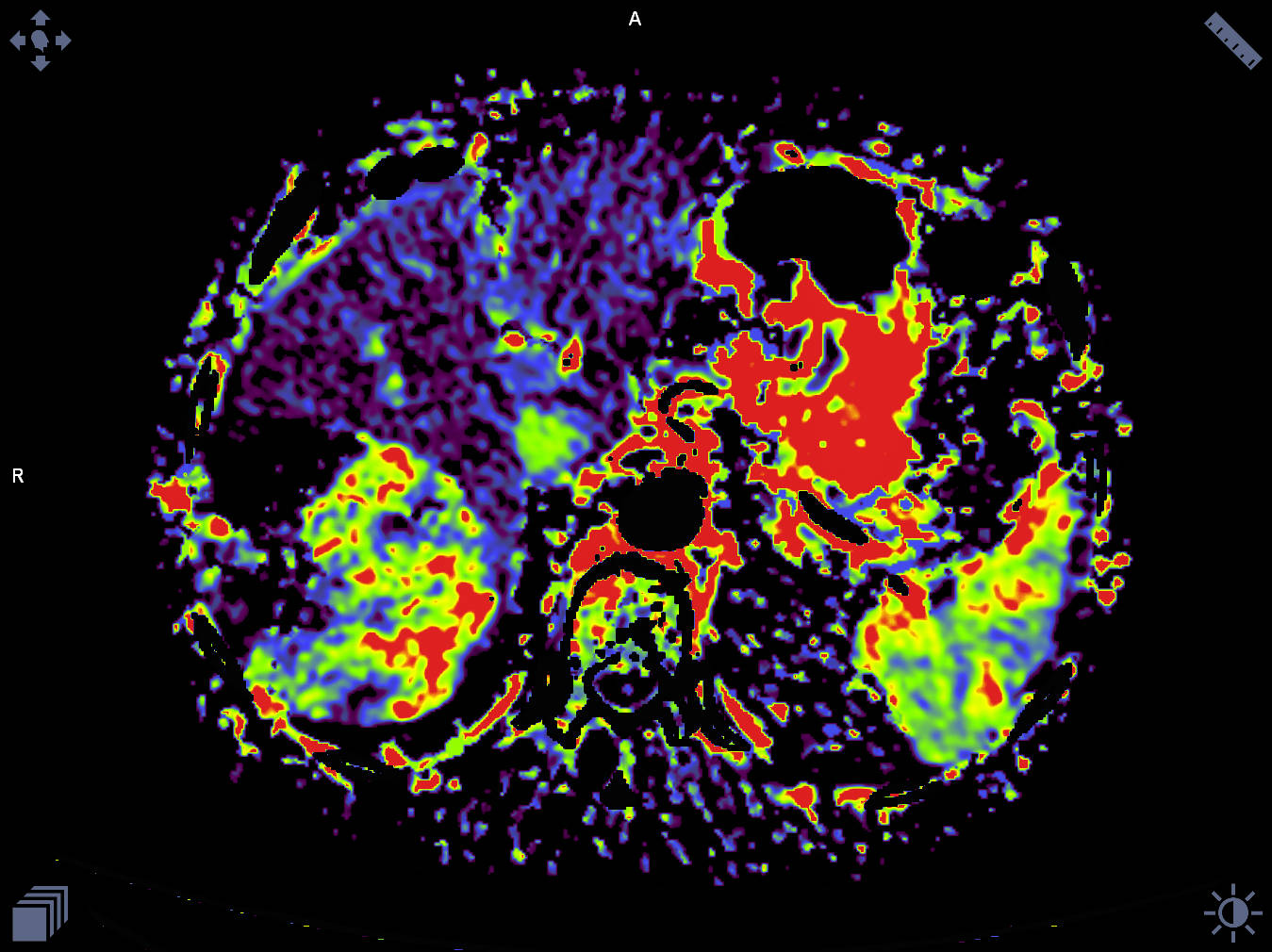


a

b

c

c
